# Two new species and two new records of fungus-feeding Phlaeothripinae from China (Thysanoptera, Phlaeothripidae)

**DOI:** 10.3897/zookeys.694.14616

**Published:** 2017-08-29

**Authors:** Chao Zhao, Xiaoli Tong

**Affiliations:** 1 Department of Entomology, College of Agriculture, South China Agricultural University, Guangzhou 510642, China

**Keywords:** leaf litter thrips, *Mystrothrips*, new species, *Urothrips*

## Abstract

Two new species of fungivorous Phlaeothripinae, *Mystrothrips levis* **sp. n.** and *Urothrips lancangensis* **sp. n.**, are described from China. *Pentagonothrips antennalis* Haga & Okajima and *Plectrothrips bicolor* Okajima are newly recorded in China.

## Introduction

The species of fungivorous Phlaeothripinae belong to *Phlaeothrips* lineage, which are usually taken from dead branches or leaf-litter and feed on fungal hyphae (Mound 2013, Minaei 2013, Dang et al. 2014). The fungivorous Phlaeothripinae fauna of China was poorly known until 30 years ago, so that only 46 species in 18 genera of this group were reported from this country (Tong and Zhang 1989). Recently, the Chinese fungivorous Phlaeothripinae was well reviewed by Dang and Qiao (2014), and subsequently three additional species of the group were recorded from China (Zhao and Tong 2016, Tong and Zhao 2017). Up to the present, 98 species and 31 genera of fungus-feeding Phlaeothripinae are recorded from China. As a large country which is across the Palaearctic and Oriental regions, China harbors an enormous diversity of Thysanoptera, yet the thrips fauna remains poorly understood. This is especially true for the group of fungus-feeding Phlaeothripinae. During recent surveys of the thrips fauna in southern China, some species of fungus-feeding Phlaeothripinae have been collected. The aim of the present paper is to describe two new species and two newly recorded species of the group from China.

## Materials and methods

All thrips specimens were extracted by using Tullgren funnels from leaf litter, and then sorted and preserved in 90% alcohol. Examined specimens were mounted into Canada balsam using the method outlined by Zhang et al. (2006). Slide-mounted specimens were examined and photographed under ZEISS Imager A1 microscope with a digital camera attached. All specimens in this study were collected from leaf-litter unless otherwise noted. All type specimens are deposited in the Insect Collection, South China Agricultural University (**SCAU**).

## Taxonomy

### Mystrothrips levis sp. n.

Taxon classificationAnimaliaThysanopteraPhlaeothripidae

http://zoobank.org/5399B364-0B5C-4B37-A2F5-896C751EF8A6

Figs 1–8

#### Material examined

**(females and males all apterous). Holotype.** Female aptera: **CHINA**, Guangdong: Guangzhou City, South China Botanical Garden (23Guangdong: Guan), in leaf litter of bamboo, 9.viii.2014 (Chao Zhao).

**Paratypes**. 8 females 2 males, collected with holotype; 5 females 1 male, the same locality but collected on 20.xi.2015 (Chao Zhao).

#### Description.

**Figures 1–8.**
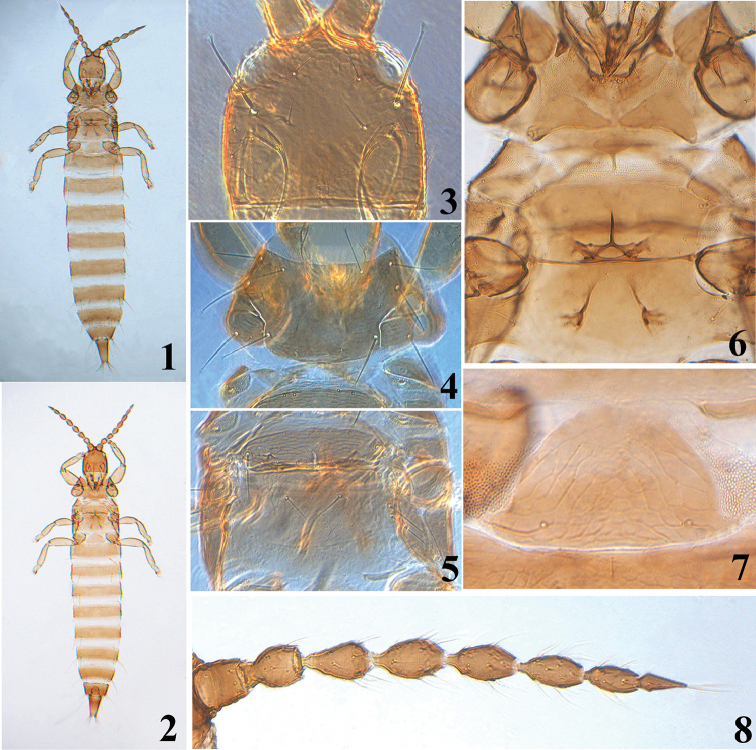
*Mystrothrips levis* sp. n. **1** female **2** male **3** head **4** pronotum **5** meso- and metanotum **6** ventral view of thorax **7** pelta **8** antenna.

**Female aptera** (Fig. 1). Body and antennae uniformly brown. All legs yellowish brown except for tarsi yellow.

*Head* (Fig. 3) almost as long as broad; dorsal surface smooth medially but with polygonal reticulation between eyes and faint reticulation laterally and posteriorly; cheeks slightly convex and weakly constricted just behind eyes; eyes small and bulging, slightly less than 1/4 of head length; postocular setae long and expanded at apex, approximately half of head length; ocelli absent; postocellar setae and mid-dorsal setae as long as eyes, pointed at apex. Antennae 8-segmented (Fig. 8), approximately 2.5 times as long as head; segments III and IV with two and three sense cones, respectively; segments II–V sculptured; segment VIII strongly constricted at base. Maxillary stylets nearly retracted to postocular setae, approximately one-third of head width apart medially.

*Pronotum* dorsal surface almost smooth but with faint transverse lines anteriorly; notopleural sutures complete; five pairs of major setae long and expanded (Fig. 4). Basantra present but small; ferna and prospinasternum well-developed. Mesonotum sculptured with distinctly transverse reticulation, and a pair of well-developed long lateral setae expanded at apex; mesopresternum eroded medially, divided into two small irregular lateral plates. Metanotum weakly sculptured with polygonal reticulation, a pair of long and acute setae situated medially (Fig. 5); meso- and metasternum smooth; metathoracic sternopleural sutures absent (Fig. 6); mesoeusternum anterior margin entire, mesothoracic furcae united together medially, but metathoracic furcae separated. Fore tarsal tooth absent.

*Pelta* nearly semicircular in shape with short lateral lobes, distinctly reticulate, a pair of campaniform sensilla present (Fig. 7); abdominal tergites weakly sculptured with reticulation, without developed sigmoid wing retaining setae; S1 and S2 setae on tergites II–VIII well-developed, long and expanded at apex; S1 setae on tergite II much longer than S2, approximately 3.0 times as long as S2; tergites III–VII with S1 and S2 setae subequal in length; S1 on tergite VIII approximately 0.7 times as long as S2; tergite IX with S1 and S2 setae subequal in length, longer than tube, weakly pointed or blunt at apex; abdominal sternites II–VIII with a transverse row of 8–16 discal setae medially, each sternite bearing two pairs of long and pointed setae arising in front of posterior margin. Tube approximately 4/5 of head length; anal setae shorter than tube.

***Measurements* (holotype female in microns).** Distended body length 2030. Head length 190, width 185; eyes length 50; postocular setae length 75. Antennae length 470, segments I–VIII length (width) as follows: 49(43); 53(41); 68(32); 67(34); 65(34); 61(30); 54(24); 52(12). Pronotum median length 140, width across median part 310; length of major setae: pronotum anteromarginal setae 48, anteroangular setae 68, midlateral setae 90, posteroangular setae 85, epimeral setae 80. Metanotum median setae 40. Pelta length 100, width at base 170. Abdominal tergite IX S1 setae length 190, intermediate setae length 85, S2 length 190. Tube length 155, width at base 93, at apex 42; anal setae length 135.

**Male aptera** (Fig. 2): Similar to apterous female in color and structure but smaller. Fore tarsal tooth present; tergite IX with S1 setae approximately 3.0 times as long as S2; sternites without pore plate.

***Measurements* (paratype male in microns).** Distended body length 1620. Head length 160, width 155; eyes length 35; postocular setae length 70. Antennae length 375, segments I–VIII length (width) as follows: 34(36); 44(29); 50(32); 50(31); 55(28); 50(25); 45(20); 45(13). Pronotum median length 125, width across median part 260; length of major setae: pronotum anteromarginal setae 60, anteroangular setae 45, midlateral setae 73, posteroangular setae 66, epimeral setae 63. Metanotum median setae 30. Pelta length 65, width at base 110. Abdominal tergite IX setae S1 length 145, intermediate setae length 60, S2 length 45. Tube length 130, width at base 80, at apex 32; anal setae length 110.

#### Distribution.

China (Guangdong).

#### Etymology.

The specific epithet, *levis*, is from the Latin adjective, meaning “smooth”, and refers to the dorsal surface of head and pronotum which are largely smooth. In contrast, most species of this genus are sculptured with distinct polygonal reticulation on head and pronotum.

#### Remarks.

Of the seven species worldwide listed in the genus *Mystrothrips* (ThripsWiki 2017), two are recorded from China. *M. longantennus* Wang, Tong & Zhang is from southern China, and *M. flavidus* Okajima is widespread from China (Guangxi, Guangdong and Taiwan) to Japan (Okajima 2006, Wang et al. 2008, Dang and Qiao 2014). The new species is most similar to *M. flavidus* in color and structure, but it can be distinguished from the latter by (1) head and pronotum largely smooth (vs sculptured with polygonal reticulation entirely in *M. flavidus*); (2) antennae uniformly brown (versus antennae segments I and II distinctly lighter than remaining segments in *M. flavidus*); (3) pelta semicircular with short lateral lobes (vs broadly trapezoidal in *M. flavidus*); (4) S1 and S2 setae on abdominal tergite IX much longer than tube (vs shorter than tube in *M. flavidus*).

### Pentagonothrips antennalis

Taxon classificationAnimaliaThysanopteraPhlaeothripidae

Haga & Okajima

Figs 9 , 10

Pentagonothrips antennalis Haga & Okajima, 1979: 147.

#### Material examined.

**CHINA**, Hunan: 2 females and 1 male, Zhuzhou City, Yanling County, Shennong Valley (26°29'N, 114°01'E), in leaf litter, 16. ix. 2014 (Chao Zhao). **Hubei**: 1 male, Huanggang City, Yingshan County, Taohuachong (30°99'04"N, 116°02'76"E), in leaf litter, 23.iv.2014 (Chao Zhao).

#### Diagnosis.

Dorsal surface of body entirely reticulate; head longer than width, cheeks distinctly incut behind eyes; postocular setae well-developed with expanded at apex. Antennae 7-segmented, morphological segments VII and VIII fused with an incomplete suture, segments III and IV with two and three sense cones, respectively. Maxillary stylets short, wide apart. Pronotum with five pairs of well-developed major setae, strongly expanded at apex. Basantra absent. Fore tarsal tooth present in both sexes. Pelta transverse, shaped as a squashed ellipse, with distinctly polygonal reticulation. Sternite VIII with pair of stout or leaf-like posteromarginal setae submedially. Tube short than head. Pore plate of male absent.

#### Distribution.

China (Hunan, Hubei); Japan.

#### Remarks.

The monobasic genus, *Pentagonothrips*, was originally established from Japan (Haga and Okajima 1979, Okajima 2006). *P. antennalis* is here recorded from China for the first time. This species is closely related to the species of the genus *Mystrothrips* Priesner in shape and structure. However, it can be separated from *Mystrothrips* by the following characters: Antennae 7-segmented; mesopresternum weak and membranous; anterior margin of mesoeusternum with a longitudinal median division (mesoeusternum anterior margin entire in *Mystrothrips*); mesothoracic furcae closely fused together medially as well as metathoracic furcae joined together medially (but metathoracic furcae separated in *Mystrothrips*).

**Figures 9–12.**
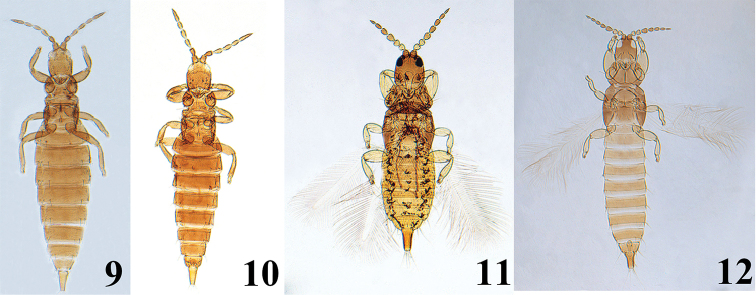
*Pentagonothrips antennalis* Haga & Okajima: **9** female **10** male *Plectrothrips bicolor* Okajima: **11** female **12** male.

### Plectrothrips bicolor

Taxon classificationAnimaliaThysanopteraPhlaeothripidae

Okajima

Figs 11 , 12

Plectrothrips bicolor Okajima, 1981: 313.

#### Material examined.

**CHINA**, Guangdong: 1 female, Guangzhou City, Arboretum of South China Agricultural University (23°09'N, 113°21'E), in leaf litter, 20.xi.2004 (Jun Wang); 1 male, Guangzhou City, Dafushan Forest Park (22°57'N, 113°18'E), in leaf litter of *Litchi chinensis*,17.iv.2016 (Chao Zhao).

#### Diagnosis.

Body bicolored, yellow and brown. Head, thorax and tube brown, abdomen yellowish brown; all legs yellow; antennal segments II and III yellow, remaining segments brown. Head longer than broad, dorsal surface smooth except weakly sculptured posterolaterally. Antennal segments III and IV with two and three sense cones, respectively, segment VI with two sense cones. Maxillary stylets short, maxillary bridge developed and arched. Pronotum smooth, surrounded by stippled membrane with a distinct median longitudinal line. Metanotum with longitudinal striae medially. Mid tibia and hind tibia with one and two apical spur-like stout setae, respectively. Forewing parallel-sided with seven duplicated cilia. Pelta irregularly triangular with slender lateral lobes and a pair of campaniform sensilla. Abdominal tergites II–VII each with a pair of wing retaining setae; sternites V–VII with a pair of worm-like reticulate areas in both sexes; tergite IX S1 and S2 setae pointed, S1 setae longer than S2 but shorter than tube; tergite IX in male with a small median projection on posterior margin.

#### Distribution.

China (Guangdong); Japan; Indonesia.

#### Remarks.

This genus now includes 32 species in the world (ThripsWiki 2017), of which three species have been reported from China (Dang et al. 2014). *P. bicolor*, originally described from Japan and Indonesia (Okajima 1981, 2006), is here newly recorded from mainland China. This species is extracted from leaf litter by using Tullgren funnels in the present study. In contrast, most species of the genus are usually collected under bark of decayed trees.

### Urothrips lancangensis sp. n.

Taxon classificationAnimaliaThysanopteraPhlaeothripidae

http://zoobank.org/B5B5318F-4ADA-49EE-928F-9C8CA9299354

Figs 13–19

#### Material examined

**(females and males all apterous).** Holotype. Female aptera, **CHINA**, Yunnan province, Pu’er City, Lancang County, Nuozhadu Nature Reserve (22°30'N,100°34'E, alt. 1840m), 5.xi.2016 (Chao Zhao).

Paratypes. 6 females, 3 males, collected with holotype.

#### Description.

**Figures 13–19.**
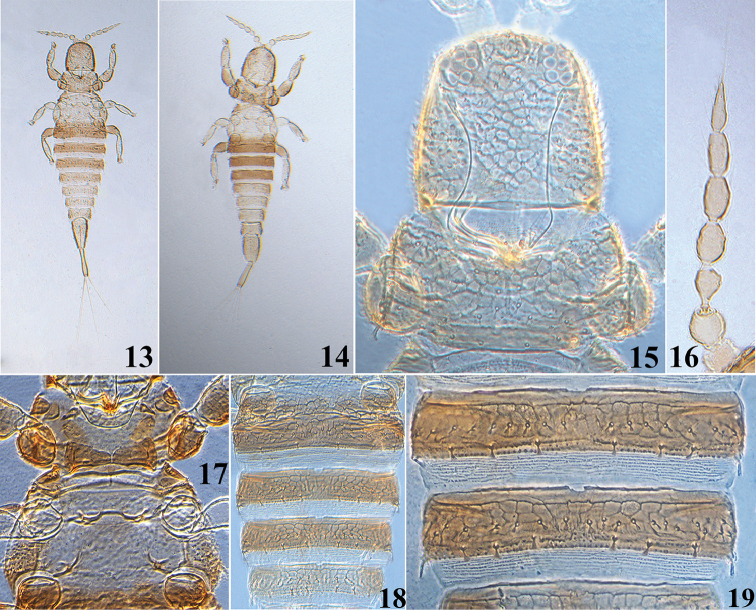
*Urothrips lancangensis* sp. n. **13** female **14** male **15** head and pronotum **16** antenna **17** pro, meso and meta sternum **18** abdominal tergites I–V 19 abdominal tergites III–IV.

**Female aptera** (Fig. 13): Body bicolored, yellow and brown; largely yellow except head, pronutum, fore and hind femora and abdominal tergites I–IV(V) brown; antennal segments II–VII tinged with light brown; tergites V–IX yellow shaded with brown laterally; tube yellow with extreme apex brown.

*Head* (Fig. 15) as long as or a little shorter than broad; head broadly rounded in front, without any prominent setae on anterior margin, weakly produced between antennae ventrally; cheeks slightly convex; dorsal surface sculptured with polygonal reticulation except tuberculate laterally and small setae weakly expended at apex. Eyes with approximately 10 facets dorsally, but absent ventrally; ocelli absent. Antennae arising ventrally, with 7 visible segments and distinct from each other (Fig. 16); segment VII without suture between morphological segments VII and VIII; segment III with one simple sense cone, situated outside of apex; IV with two simple sense cones, each approximately two-thirds as long as the segment; segments VI and VII each with one outer simple sense cone. Maxillary stylets retracted to compound eyes, approximately half of head width apart medially.

*Pronotum* transverse and rectangular (Fig. 15), approximately 2.3 times as wide as long and 0.6 times as long as head; dorsal surface sculptured with polygonal reticulation and many small setae expended at apex; epimeral setae well developed and expanded at apex. Basantra reduced to a pair of small plates laterally; ferna well developed. Meso- and metanotum with small setae expanded at apex and faintly reticulate; meta-epimeron bulging with wart-like small tubercles and one well- developed seta expanded at apex, more slender than pronotal epimeral setae. Mesopresternum complete and transverse; mesoeusternum anterior margin entire; mesothoracic furcae fused together medially, but metathoracic furcae widely separated (Fig. 17). Fore tarsus with a hook-like hamus on external margin.

*Abdomen* broadest at segment II and tapering evenly to the tube. Abdominal tergite I transverse and distinctly sculptured, closely fused to tergite II, and clearly separated from metanotum (Fig. 18); tergites II–VIII sculptured with polygonal reticulation at anterior half and with a transverse row of 10–22 short, dilated and fan-shaped setae medially, and each with three pairs of short, fan-shaped setae in front of posterior margin (Figs 18, 19); tergites III–VIII each with a pair of well-developed posterolateral setae blunt at apex; tergite IX faintly reticulate, approximately 2.5 times as long as distal wide. Tube weakly reticulate, slightly shorter than head length, constracted submedially and weakly convex near apex; tube with three pairs of anal setae; the longest lateral anal setae approximately 3.5 times as long as tube, but median dorsal pair shorter than the lateral two pairs.

Measurements (holotype female in microns). Body length 1400. Head length 180; maximum width 190. Pronotum length 110; median width 250; epimeral setae 20. Metathoracic epimeral setae 20. Abdominal tergite IX length 120, basal width 75, distal width 40. Tube length 130, basal width 22, apical width 25; anal setae 430. Antennal segments I–VIII length (width) as follows: 20(36), 28 (31), 37 (23), 39 (24), 45 (20), 40 (15), 47 (12).

**Male aptera.** (Fig. 14). Color and structure similar to apterous female, but body smaller.

**Measurements** (paratype male in microns). Body length 1050. Head length 160; maximum width 160. Pronotum length 90; median width 185; epimeral setae 13. Metathoracic epimeral setae 13. Abdominal tergite IX length 105, basal width 55, distal width 40. Tube length 115, basal width 20, apical width 22; anal setae 370. Antennal segments I–VIII length (width) as follows: 22(33), 23 (31), 32 (19), 29 (22), 33 (20), 31(17), 39(13).

#### Distribution.

China (Yunnan).

#### Etymology.

The specific epithet is named after the type locality, Lancang County, Yunnan Province, China.

#### Remarks.

There are ten species recognized in this genus (ThripsWiki 2017), of which three are recorded from China (Tong and Zhao 2017). The new species described here shares morphological affinities with *Urothrips tarai* (Stannard, 1970), particularly in the shape of antennae, but it can be differentiated from the latter by the following diagnostic characters: (1) head broadly rounded in front (vs slightly produced in *U. tarai*); (2) dorsal surfaces of head and pronotum largely sculptured with polygonal reticulation (vs head and pronotum distinctly tuberculate and without reticulation in *U. tarai*); (3) major body setae on head, pronotum, especially on abdominal tergites are stout, dilated and fan-shaped at apex (in *U. tarai*, the major body setae are fine and pointed except epimeral, meta-epimeron and abdominal tergites III–VIII posterolateral setae); (4) fore femora brown (while fore femora yellow in *U. tarai*).

## Supplementary Material

XML Treatment for Mystrothrips levis

XML Treatment for Pentagonothrips antennalis

XML Treatment for Plectrothrips bicolor

XML Treatment for Urothrips lancangensis
